# Supplementary material for: Estimating the intrinsic dimension of datasets by a minimal neighborhood information
Source: Sci Rep. 2017 Sep 22;7:12140. doi: 10.1038/s41598-017-11873-y (PMC5610237; doi:10.1038/s41598-017-11873-y)
Supplement: Supplementary file 1 — Supplementary information [file 41598_2017_11873_MOESM1_ESM.pdf]

Estimating the intrinsic dimension of datasets by a minimal neighborhood information

Elena Facco, Maria d'Errico, Alex Rodriguez, Alessandro Laio

Scuola Internazionale Superiore di Studi Avanzati (SISSA),
via Bonomea 265 - 34136 Trieste, Italy.

September 13, 2017

Supplementary information

1 Distribution of shells volumes for a homogeneous Poisson process

Let Φ be a homogeneous Poisson process in $\mathbb{R}^2$ with intensity λ (see [1] for more information about Poisson processes); in particular Φ satisfies the following properties:

i) for any disjoint Borel sets A_1 and A_2 the random variables $N(A_1)$ and $N(A_2)$ describing the number of points falling in A_1 and A_2 respectively are independent,

ii) the number of points $N(A)$ falling in a Borel set A is distributed as a Poisson variable with parameter $\lambda\mu(A)$, where $\mu(A)$ is the measure of A:

$$P(A \textit{ contains exactly n points}) \doteq P(n, A) = \frac{(\lambda\mu(A))^n}{n!}e^{-\lambda\mu(A)}$$

The intensity λ corresponds to the average density of points: $E[P(n, A)] = \lambda\mu(A)$. Moreover, the second property implies that in an infinitesimally small area dA there are no multiple points. From the definition of a Poisson process it also follows that the probability of having no points in a Borel set A (void probability) is given by:

$$P(0, A) = e^{-\lambda\mu(A)}. \tag{1}$$

Given a point o in Φ, let $d_1, d_2, ..., d_n$ be the ordered distances from o of the first n neighbours. If we define Δv_1 as the volume of the ball B_{o,d_1}, Δv_2 as the

volume of the annulus C_{r_1,r_2}, and so on we see that the distances $d_1, d_2, ..., d_n$ identify n disjoint volumes $\Delta v_1, \Delta v_2, ..., \Delta v_n$ that can be seen as the volumes 'occupied' by the neighbours. We want to find an expression for the joint probability distribution $g(\Delta v_1, \Delta v_2, ..., \Delta v_n)$. To this purpose, we start from a slightly easier problem and look for the joint probability distribution of the distances $f(d_1, d_2, ..., d_n)$.

The probability of the first distance d_1 to fall in an infinitesimally small annulus C_{r_1,r_1+dr_1} is given by the probability of having no points in the ball B_{o,r_1} and having at least one point in the annulus C_{r_1,r_1+dr_1}:

$$\begin{aligned} P(d_1 \in C_{r_1,r_1+dr_1}) =& P(N(B_{o,r_1}) = 0, N(C_{r_1,r_1+dr_1}) \geq 1) \\ =& P(N(B_{o,r_1}) = 0) P(N(C_{r_1,r_1+dr_1}) \geq 1) \\ =& P(N(B_{o,r_1}) = 0)(1 - P(N(C_{r_1,r_1+dr_1}) = 0) \\ =& e^{-\lambda r_1^2 \pi}(1 - e^{-\lambda \pi r_1 dr_1}). \end{aligned}$$

Here the second equality is due to independence property, while the last one comes from the formula for the void distribution. Since dr_1 is very small we conclude that

$$P(d_1 \in C_{r_1,r_1+dr_1}) \sim e^{-\lambda r_1^2 \pi} 2\pi\lambda r_1 dr_1. \tag{2}$$

The second step is to define the probability that the second nearest neighbour is found at a distance r_2 from o given that the first one is found at a distance r_1.

$$\begin{aligned} P(r_2 \mid r_1) \doteq& P(\text{the second nearest neighbour is at a distance } r_2 \text{ given that the first is at a distance } r_1) \\ =& P(\text{the second nearest neighbour is at a distance } r_2 \mid N(B_{o,r_1}) = 0, N(C_{r_1,r_1+dr_1}) \geq 1) \\ =& P(N(C_{r_1,r_2}) = 0, N(C_{r_2,r_2+dr_2}) \geq 1 \mid N(B_{o,r_1}) = 0, N(C_{r_1,r_1+dr_1}) \geq 1) \\ =& P(N(C_{r_1,r_2}) = 0 \mid N(B_{o,r_1}) = 0, N(C_{r_1,r_1+dr_1}) \geq 1) \cdot \\ & \cdot P(N(C_{r_2,r_2+dr_2}) \geq 1 \mid N(B_{o,r_1}) = 0, N(C_{r_1,r_1+dr_1}) \geq 1). \end{aligned}$$

We can compute separately the two terms in the product using equation 1; the first term is straightforward:

$$P(N(C_{r_1,r_2}) = 0 \mid N(B_{o,r_1}) = 0, N(C_{r_1,r_1+dr_1}) \geq 1) = e^{-\lambda\pi(r_2^2 - r_1^2)} \ ,$$

while we can write the second term as
$1 - P(N(C_{r_2,r_2+dr_2}) = 0 \mid N(B_{o,r_1}) = 0, N(C_{r_1,r_1+dr_1})),$

so that

$$P(N(C_{r_2,r_2+dr_2}) \geq 1 \mid N(B_{o,r_1}) = 0, N(C_{r_1,r_1+dr_1}) \geq 1) = 1 - e^{-\lambda\pi r_2 dr_2} \sim 2\lambda\pi r_2 dr_2$$

Finally we obtain a formula for $P(r_2 \mid r_1)$:

$$P(r_2 \mid r_1) \sim e^{-\lambda\pi(r_2^2 - r_1^2)} 2\lambda\pi r_2 dr_2.$$

Now we can compute the joint probability $P(r_1, r_2)$:

$$P(r_1, r_2) = P(r_2 \mid r_1)P(r_1) \sim e^{-\lambda\pi r_2^2}(2\lambda\pi)^2 r_1 r_2 dr_1 dr_2.$$

This result can be generalized to the third neighbour:

$$P(r_1, r_2, r_3) = P(r_3 \mid r_1, r_2)P(r_2 \mid r_1)P(r_1) \sim e^{-\lambda\pi r_3^2}(2\lambda\pi)^3 r_1 r_2 r_3 dr_1 dr_2 dr_3,$$

and so on to the nth neighbor:

$$P(r_1, r_2, ..., r_n) \sim e^{-\lambda\pi r_n^2}(2\lambda\pi)^n r_1 r_2 \cdots r_n dr_1 dr_2 \cdots dr_n,$$

so that the expression for the joint probability distribution of the distances is given by:

$$f(r_1, ..., r_n) = e^{-\lambda\pi r_n^2}(2\lambda\pi)^n r_1 r_2 \cdots r_n.$$

Now, we are interested in the distribution of volumes. The change of variables $\alpha : (r_1, r_2, ..., r_n) \longmapsto (\Delta v_1, \Delta v_2, ..., \Delta v_n)$ defined as

$$(r_1, r_2, ..., r_n) \longmapsto (\pi r_1^2, \pi(r_2^2 - r_1^2), ..., \pi(r_n^2 - r_{n-1}^2))$$

is an omeomorphism on $\mathbb{R}^2_{>0}$; let β be the inverse. If we denote by $\mid D\beta \mid$ and $\mid D\alpha \mid$ the jacobians of β and α respectively, we obtain

$$\begin{aligned} g(\Delta v_1, \Delta v_2, ..., \Delta v_n) =& f(\beta(\Delta v_1, \Delta v_2, ..., \Delta v_n)) \mid D\beta \mid_{|\Delta v_1, \Delta v_2, ..., \Delta v_n} \\ =& f(\beta(\Delta v_1, \Delta v_2, ..., \Delta v_n)) \mid D\alpha \mid^{-1}_{|\beta(\Delta v_1, \Delta v_2, ..., \Delta v_n)} . \end{aligned}$$

Now we can easily compute the jacobian of α as

$$\mid D\alpha \mid_{|r_1, r_2, ..., r_n} = \pi^n 2^n r_1 \cdots r_n = (2\pi)^n (\beta(\Delta v_1), \beta(\Delta v_2), ..., \beta(\Delta v_n)).$$

Finally, the expression for g is given by:

$$g(\Delta v_1, \Delta v_2, ..., \Delta v_n) = \lambda^n e^{-\lambda(\Delta v_1 + \Delta v_2 + ... + \Delta v_n)},$$

so that the joint distribution of volumes is exponential with parameter equal to the average density of points.

This argument can be easily generalized to $\mathbb{R}^N$.

2 A comparison between TWO-NN and DANCo

We compare our results with those obtained with DANCo [2] since, according to the analysis in [3], it seems to outperform the other estimators (a public version of DANCo algorithm is available at https://it.mathworks.com/matlabcentral/fileexchange/40112-intrinsic-dimensionality-estimation-techniques/content/idEstimation/DANCoFit.m.).
In order to test DANCo in the case of uniform hypercubes with periodic boundary conditions we modified the computation of distances in the code. First of all we analyzed the estimates of DANCo and TWO-NN on datasets with 2500 points and dimension ranging from 1 to 20. The selected datasets are hypercubes without periodic boundary conditions, hypercubes with periodic boundary conditions, Cauchy dataset and Gaussians. We embed the datasets in higher dimensional spaces through the identity map to prevent the algorithms from selecting the number of columns as an upper bound.
In the case of hypercubes without pbc (panel A) TWO-NN produces an underestimation (about 1.5 in dimension 10 and 4 in dimension 20), due to the sharp drop in density at the border. This systematic error becomes smaller and smaller when the number of points is increased. A similar but lighter effect is visible in the case of gaussian distributions (panel D): here the density changes rapidly but in a smoother fashion. We notice an underestimation of around 0.1 in dimension 10 and 3 in dimension 20. In panel B we see that considering periodic boundary conditions (and thus reproducing a most uniform environment) allows TWO-NN to estimate the ID almost correctly, with an underestimation of the order of 1 in dimension 20. In the case of Cauchy dataset (panel C) TWO-NN slightly overestimates the intrinsic dimension. As for DANCo, we notice that it slightly overestimates the dimension for the Hypercubes and for the Gaussian, while it strongly underestimates the value of the ID in the case of Cauchy dataset (the estimate for a Cauchy dataset in dimension 20 is around 13). We believe that the origin of this significant systematic error lies in the fact that DANCo estimates the ID by comparing the theoretical functions obtained in the dataset with those retrieved on uniform spheres: this strategy works well in the case of sharp boundaries but is less suitable in the presence of heavy tails.

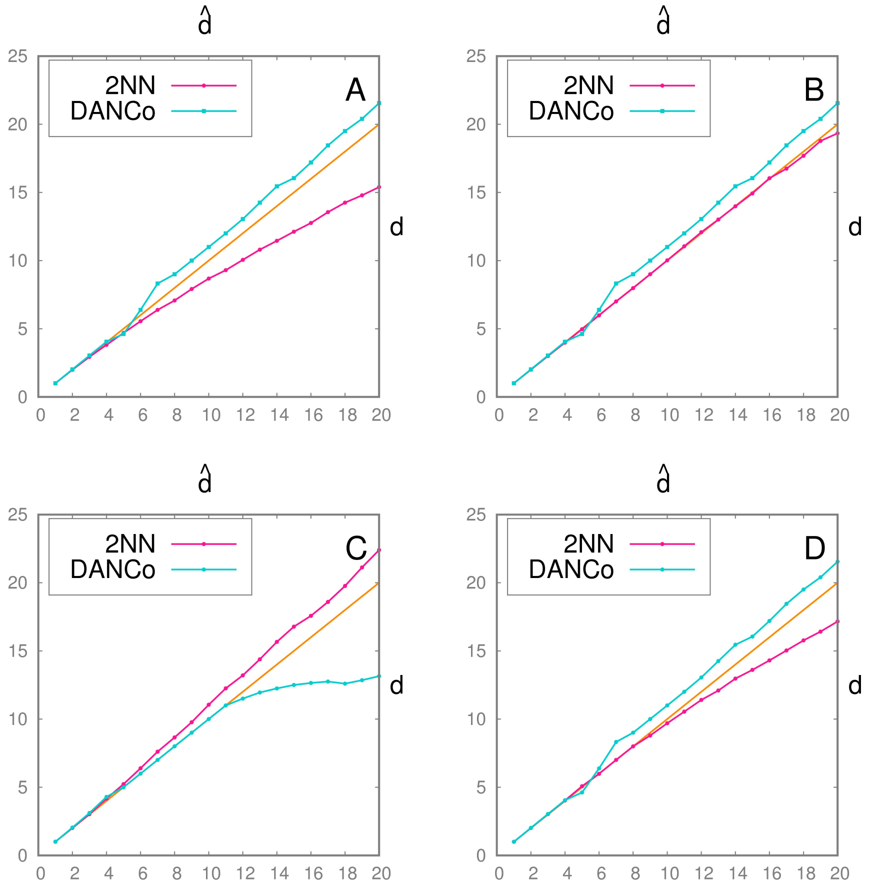

Figure 1: ID estimates for DANCo and TWO-NN on selected datasets of 2500 points. For each dimension we take as ID estimate the average over 20 instances of the dataset. On the x-axis and y-axis we represent the true dimension of the dataset d and the estimated dimension $\hat{d}$ respectively. Panel A: Hypercubes embedded in a space of dimension $d+5$ through the identity map; the test is carried out with no periodic boundary conditions. Panel B: Hypercubes embedded in a space of dimension $d+5$ through the identity map; the test this time is carried out with periodic boundary conditions. Panel C: Cauchy datasets embedded in a space of dimension $d+3$. Panel D: gaussian distributions embedded in a space of dimension $d+5$.

3 Discarding the points with highest values of μ

In Section 3 we claim that in order to make the procedure more robust we discard the 10% of the points characterized by highest values of μ from the fitting.

Indeed, outliers in the dataset display high values of μ and are able to affect the linear fitting procedure in a meaningless way. Simply cutting the very last points away from the dataset S to fit makes the procedure robust. The decision to exclude the last 10% of points is arbitrary, but the estimate of the dimension is robust respect to this threshold. In Figure 2 we see that the estimated dimension is the same for a percentage of retained points ranging from 80% to 95%, while including all of the points causes instability and underestimation. Cauchy datasets are characterized by heavy tails and so the presence of outliers is important; if we consider uniform hypercubes outliers are nearly absent and the ID estimate is not affected by the exclusion from the fit of the last points with highest μ, as we can see in Figure 3.

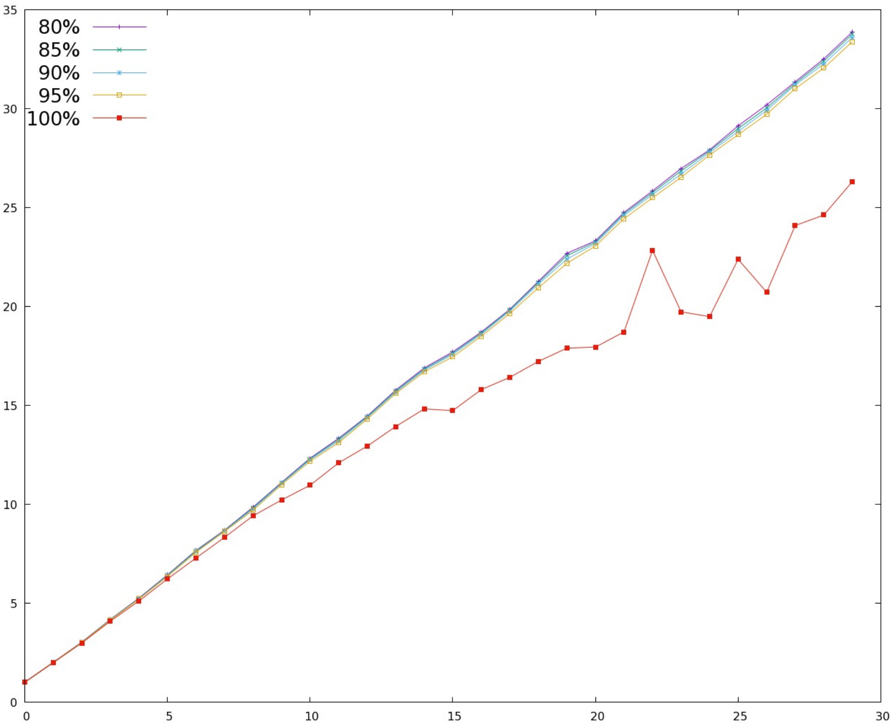

Figure 2: Estimated ID averaged over 20 samples of a Cauchy dataset (y-axis) vs real ID (x-axis) for different percentages of retained points ranging from 80% to 100%. The dimension is robust respect to the threshold of retained points, but including all of them causes instability in the measure.

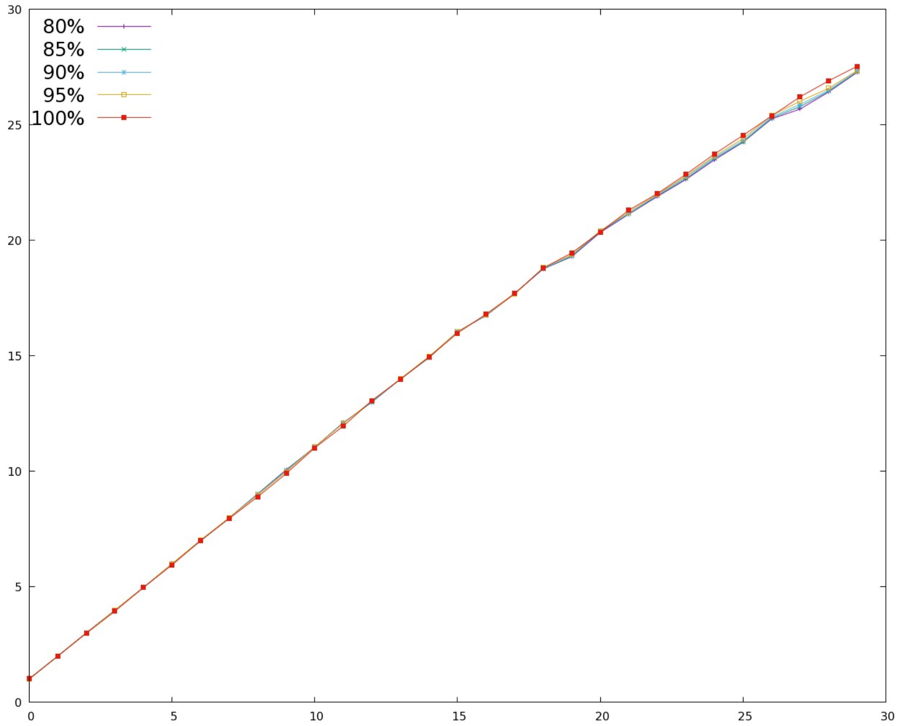

Figure 3: Estimated ID averaged over 20 samples of a uniform hypercube (y-axis) vs real ID (x-axis) for different percentages of retained points ranging from 80% to 100%. The ID estimate is not affected by the exclusion from the fit of the last points with highest μ.

4 Additional benchmark: synthetic datasets

We further tested TWO-NN on the synthetic benchmarks proposed in [3], listed in Table 1 together with their relevant features N, d, D; they include datasets characterized by high dimensionality, sharp edges, or described by a complex non-linear embedding. Datasets from M_1 to M_{13} are generated from the publicly available tool (http://www.mL.uni-saarland .de/code/IntDim/IntDim.htm) proposed by Hein and Audibert in [4]; only dataset M_8 is missing from the analysis in [3], since according to the authors it is particularly challenging for its high curvature and induces pronounced overestimates in many relevant ID estimators (see [3]). Datasets M_{P3}, M_{P6}, M_{P9} (see [5]) are interesting because the underlying manifold is characterized by a nonconstant curvature. Finally, datasets M_{N1}, M_{N2}, M_{beta} are proposed by the authors of [3] themselves. For a full description of the datasets and tools to generate them refer to [3].

Table 1: The 21 synthetic datasets proposed in [3]

Dataset	Description	N	d	D
M_1	10-dimensional hypersphere linearly embedded	2500	10	11
M_2	Affine space	2500	3	5
M_3	Concentrated figure, mistakable with a 3 dimensional one	2500	4	6
M_4	Nonlinear manifold	2500	4	8
M_5	2-dimensional helix	2500	2	3
M_6	Nonlinear manifold	2500	6	36
M_7	Swiss-Roll	2500	2	3
M_9	Affine space	2500	20	20
M_{10a}	10-dimensional hypercube	2500	10	11
M_{10b}	17-dimensional hypercube	2500	17	18
M_{10c}	24-dimensional hypercube	2500	24	15
M_{10d}	70-dimensional hypercube	2500	70	71
M_{11}	Möebius band 10-times twisted	2500	2	3
M_{12}	Isotropic Multivariate Gaussian	2500	20	20
M_{13}	1-dimensional helix curve	2500	1	3
M_{N1}	Manifold non-linearly embedded in $\mathbb{R}^{72}$	2500	18	72
M_{N2}	Manifold non-linearly embedded in $\mathbb{R}^{96}$	2500	24	96
M_{beta}	Manifold non-linearly embedded in $\mathbb{R}^{40}$	2500	10	40
M_{P3}	Manifold non-linearly embedded in $\mathbb{R}^{12}$	2500	3	12
M_{P6}	Manifold non-linearly embedded in $\mathbb{R}^{21}$	2500	6	21
M_{P9}	Manifold non-linearly embedded in $\mathbb{R}^{30}$	2500	9	30

We added to the proposed benchmarks some synthetic datasets we list and descript in Table2; since the large majority of the datasets proposed in [3] are characterized by boundaries where the density drop is very sharp, or even discontinuous, our 7 new benchmarks display a smooth behaviour at the boundaries. $C10$, $C15$, $C30$ are Cauchy datasets and $HC10$, $HC17$, $HC24$ are uniform hy-

percubes embedded through an identity map in a higher dimensional space; on the latters we test the method applying periodic boundary conditions (pbc) in order to simulate as much as possible a uniform environment.

Table 2: The 7 additional datasets

Dataset	Description	N	d	D
$C10$	10-dimensional cauchy dataset linearly embedded in $\mathbb{R}^{15}$	2500	10	15
$C15$	15-dimensional cauchy dataset linearly embedded in $\mathbb{R}^{20}$	2500	15	20
$C30$	30-dimensional cauchy dataset linearly embedded in $\mathbb{R}^{35}$	2500	30	35
$M8$	12-dimensional manifold embedded in $\mathbb{R}^{72}$	2500	12	72
$HC10$	10-dimensional hypercube linearly embedded in $\mathbb{R}^{15}$	2500	10	15
$HC17$	17-dimensional hypercube linearly embedded in $\mathbb{R}^{22}$	2500	17	22
$HC24$	24-dimensional hypercube linearly embedded in $\mathbb{R}^{29}$	2500	24	29

As suggested in [3] we generated 20 instances of each dataset and averaged the achieved results; The result of the tests is summarized in Figure 4. We omit to display the measure for dataset M_{10d} since its ID is 70, and estimating the dimension of such datasets is beyond the intentions of TWO-NN (indeed as we expect we undergo a strong underestimation of 41 in this case).

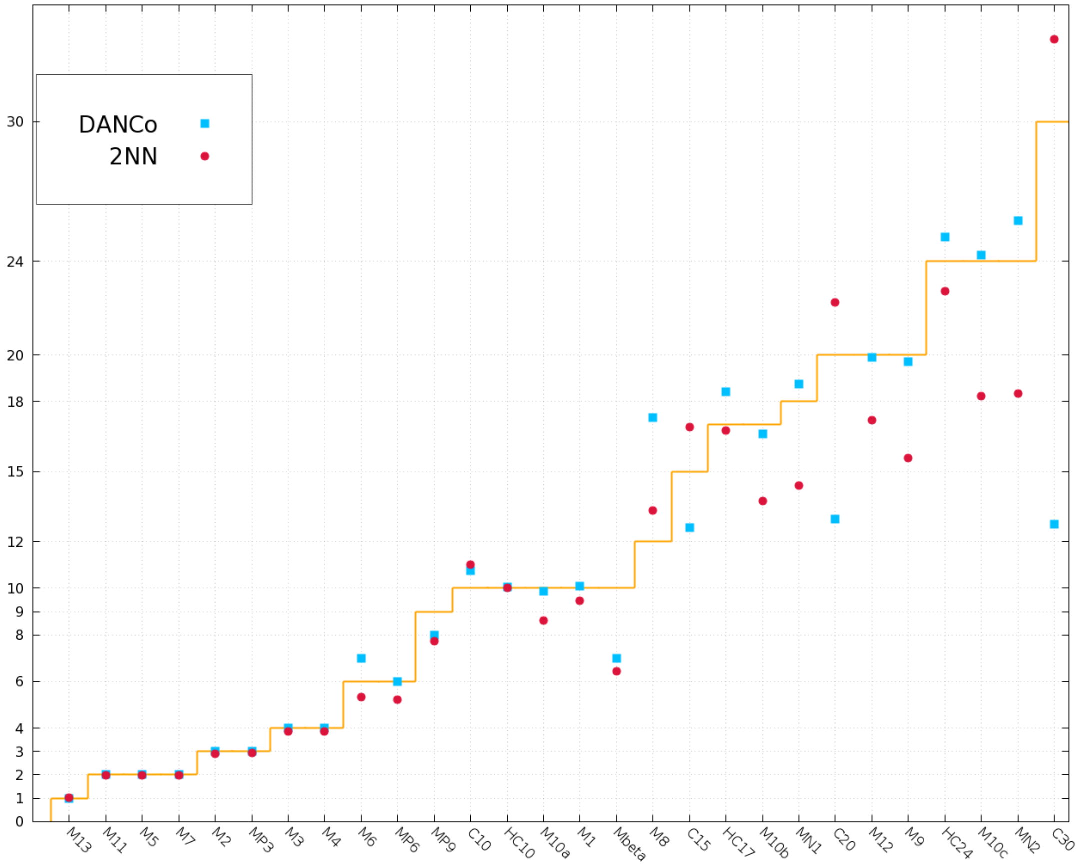

Figure 4: ID estimates for DANCo and TWO-NN on 20 selected datasets of 2500 points described in Table 1 plus 7 additional datasets described in Table 2 . For each dimension we take as ID estimate the average over 20 instances of the dataset. On the x-axis and y-axis we represent the true dimension of the dataset d and the estimated dimension $\hat{d}$ respectively.

References

[1] D. Moltchanov, "Distance distributions in random networks," *Ad Hoc Networks*, vol. 10, no. 6, pp. 1146–1166, 2012. DOI 10.1016/j.adhoc.2012.02.005

[2] C. Ceruti, S. Bassis, A. Rozza, G. Lombardi, E. Casiraghi, and P. Campadelli, "Danco: An intrinsic dimensionality estimator exploiting angle and norm concentration," *Pattern recognition*, vol. 47, no. 8, pp. 2569–2581, 2014. DOI 10.1016/j.patcog.2014.02.013

[3] P. Campadelli, E. Casiraghi, C. Ceruti, and A. Rozza, "Intrinsic dimension estimation: Relevant techniques and a benchmark framework," *Mathematical Problems in Engineering*, vol. 2015, 2015. DOI 10.1155/2015/759567

[4] M. Hein and J.-Y. Audibert, "Intrinsic dimensionality estimation of submanifolds in r d," in *Proceedings of the 22nd international conference on Machine learning*, pp. 289–296, ACM, 2005. DOI 10.1145/1102351.1102388

[5] M. Brito, A. Quiroz, and J. E. Yukich, "Intrinsic dimension identification via graph-theoretic methods," *Journal of Multivariate Analysis*, vol. 116, pp. 263–277, 2013. DOI 10.1016/j.jmva.2012.12.007
